# Evaluation of 3D-Printing Scaffold Fabrication on Biosynthetic Medium-Chain-Length Polyhydroxyalkanoate Terpolyester as Biomaterial-Ink

**DOI:** 10.3390/polym13142222

**Published:** 2021-07-06

**Authors:** Anuchan Panaksri, Nuttapol Tanadchangsaeng

**Affiliations:** College of Biomedical Engineering, Rangsit University, 52/347 Phahonyothin Road, Pathum Thani 12000, Thailand; anuchan.p59@rsu.ac.th

**Keywords:** medium-chain-length polyhydroxyalkanoate, biosynthesized terpolyester, 3D-printing, biomaterial-ink, scaffold

## Abstract

Currently, the selection of materials for tissue engineering scaffolds is still limited because some tissues require flexible and compatible materials with human cells. Medium-chain-length polyhydroxyalkanoate (MCL-PHA) synthesized in microorganisms is an interesting polymer for use in this area and has elastomeric properties compatible with the human body. MCL-PHAs are elastomers with biodegradability and cellular compatibility, making them an attractive material for fabricating soft tissue that requires high elasticity. In this research, MCL-PHA was produced by fed-batch fermentation that *Pseudomonas Putida* ATCC 47054 was cultured to accumulate MCL-PHA by using glycerol and sodium octanoate as carbon sources. The amounts of dry cell density, MCL-PHA product per dry cells, and MCL-PHA productivity were at 15 g/L, 27%, and 0.067 g/L/h, respectively, and the components of MCL-PHA consisting of 3-hydroxydecanoate (3HD) 64.5%, 3-hydroxyoctanoate (3HO) 32.2%, and 3-hydroxyhexanoate (3HHx) 3.3%. The biosynthesized MCL-PHA terpolyester has a relatively low melting temperature, low crystallinity, and high ductility at 52 °C, 15.7%, and 218%, respectively, and considering as elastomeric polyester. The high-resolution scaffold of MCL-PHA terpolyester biomaterial-ink (approximately 0.36 mm porous size) could be printed in a selected condition with a 3D printer, similar to the optimum pore size for cell attachment and proliferation. The rheological characteristic of this MCL-PHA biomaterial-ink exhibits shear-thinning behavior, leading to good shape fidelity. The study results yielded a condition capable of fabricating an elastomer scaffold of the MCL-PHA terpolyester, giving rise to the ideal soft tissue engineering application.

## 1. Introduction

The scaffolds currently studied were developed to respond to various specific-tissued properties, such as mechanical support and porosity formation [1]. Nowadays, scaffold techniques are available to make them more effective in molding and material selection [2,3]. 3D printing is the processing of a workpiece by injecting a material into the shape designed by a computer program [4]. The injected material was extruded by a pressure-based fabrication technique that has been rendered in a viscoelastic state [5]. The advantage of this technique is that it is easy to create minuscule pores of the scaffold [6]. However, the scaffold formation must have cellular compatibility; thus, the employed material must be non-cytotoxic and conformed to the created tissue [7]. These suitable materials could be biopolymer materials such as polycaprolactone [8], polylactic acid [9], and polyhydroxyalkanoates [10], which can be employed as a biomaterial-ink [11,12].

Medium-chain-length polyhydroxyalkanoates (MCL-PHAs) belong to the group of polyhydroxyalkanoates (PHAs), which are polyesters that accumulate inside microbial cells and can be synthesized from renewable resources [13]. The PHAs can be divided according to the number of carbon atoms within the molecular structure; short-chain length polyhydroxyalkanoates (SCL-PHAs) have 3 to 5 carbon atoms, and medium-chain-length polyhydroxyalkanoates (MCL-PHAs) have 6 to 14 carbon atoms. These polyesters are biodegradable and biocompatible [14]. In addition, MCL-PHAs are elastomers with high flexibility and elongation, different from SCL-PHAs, which are hard and brittle [15]. With a wide range of MCL-PHA features, it has attracted attention to medical applications such as artificial heart valves, artificial blood vessels, artificial skin, and drug delivery systems [16].

Based on the above information, we further highlighted the possibilities of using an MCL-PHA as a biomaterial-ink in a 3D printing application. MCL-PHAs are highly flexible and degradable, which there are very few elastomer types with these properties. Therefore, it has gained attention in studies of a cardiac tissue scaffold [17] and other biomedical applications with elastomeric properties and cell compatibility. However, MCL-PHAs are rubber-like materials, making it difficult and challenging to form porous scaffold structures. In this research, the 3D printing technique was applied to fabricate a scaffold using a biosynthesized MCL-PHA that examined all the polymer characteristics. In addition, the obtained MCL-PHA has been evaluated by its capability to form porous scaffold using 3D printing techniques for optimization conditions for further utilization and application.

## 2. Materials and Methods

### 2.1. Bacteria and Culture Condition

A laboratory mutant strain of *Pseudomonas putida* ATCC47054 was used in this study. The strain was adapted in a glycerol and sodium octanoate-productive environment and effectively formed MCL-PHAs. Typically, the strain was maintained in 45% glycerol kept at –80 °C. An inoculum of *Pseudomonas putida* ATCC47054 was prepared by aseptically transferring the cells from –80 °C into 5 mL of nutrient broth medium in a test tube and incubated for 24–48 h at 30 °C and then subcultured on nutrient agar for the single colony. The cells taken from an only settlement were further cultivated in a 500-mL flask containing 200 mL of a mineral solution supplemented with 20 g/L glycerol and 1 g/L sodium octanoate and shaken at 30 °C and 200 rpm for 36–40 h on a rotary shaker. The mineral solution contained the following (per liter): 1.2 g of NaH_2_PO_4_, 0.5 g of MgSO_4_·7H_2_O, 2 g of (NH_4_)_2_SO_4_, 7.34 g/L K_2_HPO_4_, and 1 mL of trace element solution, which contained 700 mM Fe(NH_4_)SO_4_, 17 mM ZnSO_4_·7H_2_O, 25 mM MnCl_2_·4H_2_O, 8 mM CuSO_4_·5H_2_O, 7.2 mM NaB_4_O_2_·10H_2_O, and 8.3 mM NaMoO_4_·2H_2_O. The flask culture was repeated two times to obtain highly active cells as the inoculum for fermentation in a bench-top bioreactor.

The fermentation process was conducted in the MDFT-N-5L Bioreactor (B.E.Marubishi, Pathumthani, Thailand) containing the 2.5-L mineral solution described above, with a fermentation duration of 60 h. The temperature, pH, and dissolved oxygen probes were used to monitor and control the fermentation. Culture conditions were maintained at 30 °C and pH 6.8. Agitation speeds were 200 rpm or above to control the dissolved oxygen at 10% of air saturation. Cultures were inoculated with a 200-milliliter seed flask culture, as mentioned above. The fed-batch fermentation process was added with carbon sources of glycerol, sodium octanoate, and nitrogen source of ammonia during fermentation for control pH that reduces due to bacteria activity. The glycerol was added at the beginning of the fermentation of 20 g/L. In the 20 to 60 h period, a glycerol:octanoate solution (7:3 ratio) was added to biosynthesize PHA in the growth phase and the nutrient limiting–PHA accumulation phase, at which sodium hydroxide was replaced with 15% ammonium solution for approximately 40-h fermentation [18,19]. Aliquots of 10–15 mL culture medium were collected for analysis of OD_600_ and cell dry weight. After 60-h cultivation, the cells were harvested, centrifuged, lyophilized, and used for the determination and characterization of MCL-PHA.

### 2.2. Quantification of MCL-PHA by Gas Chromatography

About 50 mg of lyophilized cells were added with 2 mL of chloroform and 2 mL of 3% H_2_SO_4_ (*v*/*v*) in methanol supplemented with 10 mg/mL benzoic acid as the internal standard and then heated at 100 °C for 4 h with mixing at 30 min intervals. After that, the tube of the mixture was placed at room temperature and left to cool down overnight. Then, 2 mL of distilled water was added, mixed vigorously by vortex for 4 min, and the tube was left to stand overnight. The chloroform layer was used for the determination of MCL-PHA in milligram by comparing with the sodium octanoate standard curve resulted from gas chromatography data analysis, carried out on an Agilent 7890A GC gas chromatography instrument (Agilent Technologies, Wilmington, DE, USA). The percentage of MCL-PHA content per dry cell weight (%*w/w*) and MCL-PHA yield (mg/L) were calculated as previously described [20].

### 2.3. Extraction and Characterization of MCL-PHA

The lyophilized cells were added with chloroform at a ratio of 15 mL of chloroform per 1 g of the lyophilized cells. First, the cells and chloroform were mixed by using a magnetic stirrer, and the suspension was then placed on the heat box at 60 °C overnight; this step was repeated twice. After that, the suspension was filtered through Whatman no. 1 filter paper (GE Healthcare Life Sciences, Marlborough, MA, USA), the filtrate was dropped into a beaker contained with 60 mL of methanol until the polymer was precipitated in a slurry state. Then, methanol was removed by filtering through filter paper and, subsequently, evaporated at 50 °C. Next, polymer films of approximately 100 µm thickness were prepared by casting PHA-chloroform solutions onto Teflon Petri dishes and allowing the solvent to evaporate for one week to reach a constant weight. The solvent-casted films were further aged in a vacuum at 50 °C for at least one week and, subsequently, stored at room temperature for a month prior to use in further characterization.

The PHA polymer was characterized by using a 600 MHz Bruker Avance III HD NMR (nuclear magnetic resonance) spectrometer with a Bruker Ascend^TM^ 600 (Bruker, Billerica, MA, USA). MCL-PHA sample was dissolved in CDCl_3_ (25 mg/mL) via gentle mixing and heating and analyzed with ^1^H and ^13^C NMR spectroscopy to find the chemical structure information and the monomer composition. In addition, attenuated total reflectance Fourier transform infrared (ATR-FTIR) spectroscopy was carried out via a Nicolet 6700 FTIR spectrometer (Nicolet Instrument Corporation, Madison, WI, USA) operating with a wavelength range of 500 to 4000 cm^−1^ and at a resolution of 2 cm^−1^ [21].

### 2.4. Thermal Property and Crystallinity Analysis

Differential scanning calorimetry (DSC) analysis of MCL-PHA’s (4 mg) film sample was performed using METTLER TOLEDO DSC 3+ (Mettler-Toledo AG, Schwerzenbach, Switzerland). Scanning was conducted under a nitrogen flow of 50 mL/min from –70 to 200 °C at a heating and cooling rate of 10 °C/min. The melting temperature (*T*_m_), enthalpy of fusion (Δ*H*_m_), and glass transition temperature (*T*_g_) were determined from the DSC thermogram. In addition, the crystallinity (*X*_c_) of the PHA samples was determined from the following Equation (1) :

(1) $$X_{c}=\frac{\Delta H_{m}}{\Delta H_{PHB}^{0}}$$

where Δ*H*_m_ is the measured enthalpy of the MCL-PHA sample melting, and $\Delta H_{PHB}^{0}$ is the theoretical enthalpy of a pure crystal melting of polyhydroxybutyrate (PHB) homopolymer (146 J/g), which have the highest crystallinity value among PHA polyesters [22,23].

The crystallinity analysis of MCL-PHA film was performed using a D8 ADVANCE X-ray diffractometer (Bruker, Billerica, MA, USA) in the 2θ range 4–60° at a scan speed of 2.0°/min. The degree of crystallinity (*X*_c_) of the sample was calculated from diffracted intensity data using Vonk’s method [24].

### 2.5. Mechanical Property Testing

The mechanical properties of MCL-PHA films were evaluated using a a universal test machine (TestResources, Shakopee, MN, USA) with a 5 kg load cell equipped with tensile grips. The film specimen (40 × 10 × 0.15 mm^3^) was tested with a pulling speed of 10 mm/min. The tensile testing data were collected for analysis of various mechanical properties (repeat three times).

### 2.6. Fabrication of MCL-PHA Scaffolds with a 3D Printer

**Figure 1:**
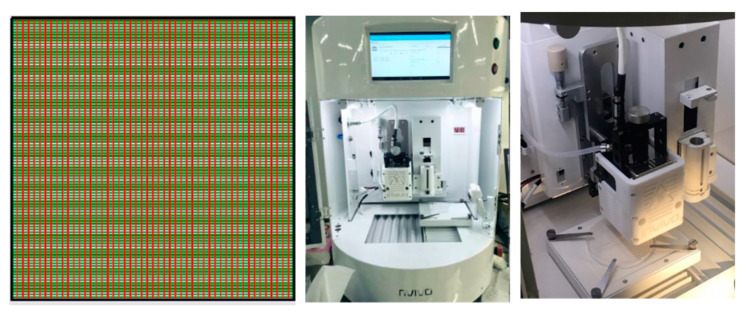
Grid scaffold pattern (top view) designed and produced for 3D-printing technique using the Rokit Invivo Multi-Use Hybrid Bio 3D Printer.

The optimization of the printing condition was performed before the scaffold was fabricated. The parameters used in the optimization are temperature and pressure, which were tested to find the suitable filament size. MCL-PHA scaffold was created by a Rokit Invivo Multi-Use Hybrid Bio 3D Printer (ROKIT HEALTHCARE, Seoul, Korea) for the determination of the 3D-printing capability, as shown in Figure 1. The scaffold was a 2 × 2 cm^2^ square with a pore area of 1 and 2 mm^2^, respectively. The 3D-printing was divided into the following 2 types: rough printing (2 × 2 mm^2^ pores) for finding the suitable condition and fine printing (1 × 1 mm^2^ pore) for a recheck of the optimal condition. Subsequently, we performed two conditions with the same melting temperature and printing speed at 130 °C and 3 mm/s. Both conditions were varied in different pressures, which one condition applied with 300 kPa and the other condition applied with 450 kPa. The printability was evaluated by the diameter measurement of the strand thickness and pore size. The strand thickness should be close to the designed fiber size and the printhead size. The scaffold obtained by 3D printing was calculated to determine printing accuracy [25]. The printing accuracy was determined by comparing the printed scaffold area and the design by the Image J software (www.imagej.nih.gov, accessed on 1 May 2021). The percentage of printing accuracy (% accuracy) was calculated using the following Equation (2):

(2) $$\%\;Accuracy=1-\frac{\left|a-b\right|}{a}\times 100$$

where *a* is the area of designed scaffold and *b* is the area of the printed scaffold that was measured using ImageJ.

### 2.7. Rheological Characterization

A rheological test (Flow test) was performed with the ARES-G2 rheometer (TA Instruments, New Castle, DE, USA). All the measurements were carried out with a parallel plate geometry (25 mm in diameter) at 130 °C. The flow test employed the shear rates of 1 to 500 s^−1^ and gave rise to the results of viscosity and shear stress. Temperature sweeps were conducted at different temperatures (60 to 160 °C) with constant shear rates (18 s^−1^). The temperature sweep test was set to plot viscosity values relative to temperature.

## 3. Results and Discussion

### 3.1. Biosynthesis of MCL-PHA Polyester by Fed-Batch Fermentation

**Figure 2:**
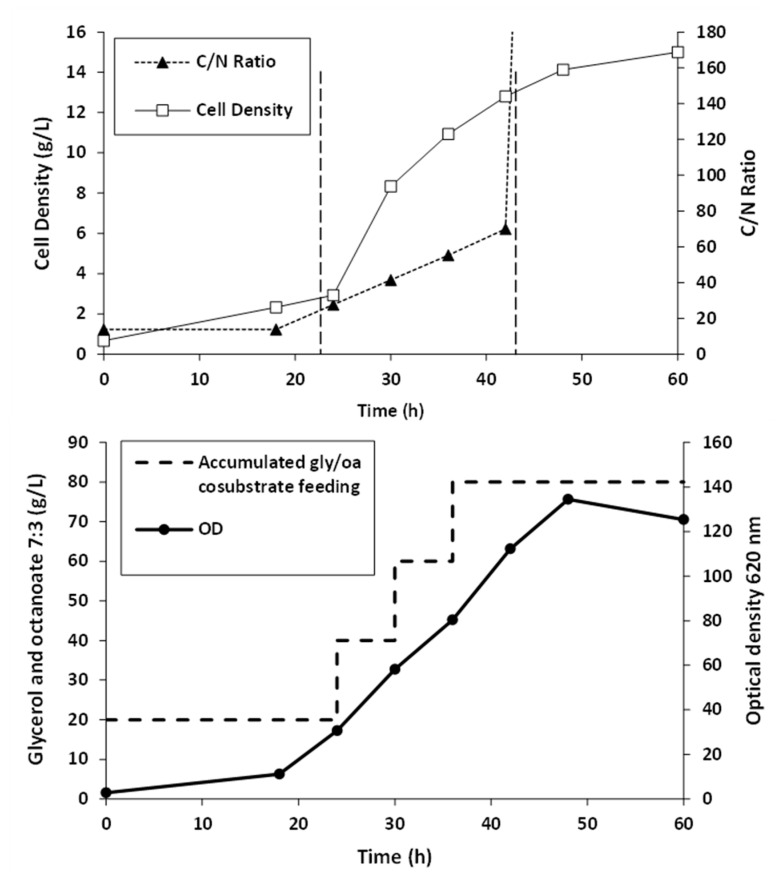
Time courses of MCL-PHA biosynthesis: cell growth, substrate, and nutrient control, in fed-batch of *Pseudomonas putida* ATCC47054 cultured by fed-batch fermentation in a 5-L bioreactor for 60 h using glycerol/octanoate co-substrates.

**Figure 3:**
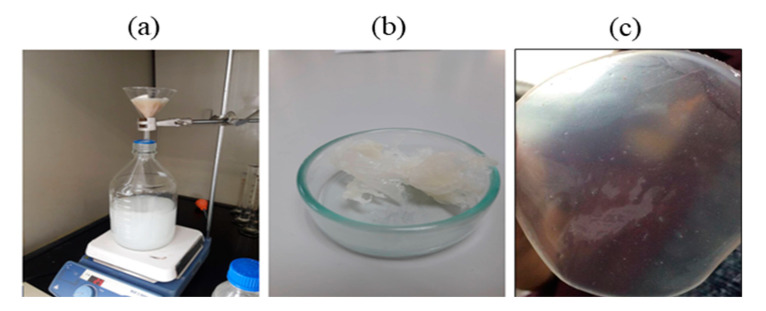
The process of extracting MCL-PHA from the dry cells using chloroform-methanol (**a**). MCL-PHA obtained from extraction (**b**). MCL-PHA sample (**c**).

Fed-batch fermentation of *Pseudomonas putida* ATCC47054 was performed in a 5 L bioreactor containing 2.5 L mineral solution supplemented with glycerol as the carbon source and sodium octanoate as the MCL-PHA precursor. The culture consisted of an initial cell growth phase on glycerol under nutrient-rich conditions, followed by PHA formation from glycerol and octanoate under nutrient limitation. After 60 h of cultivation, the cells were harvested, measured OD 620 nm, cell density, and calculated the C/N ratio used for fermentation, as shown in Figure 2. The lag phase, log phase, and stationary phase were at 0–22 h, 22–42 h, and 42–60 h. The results show that limiting the amount of nitrogen by replacing ammonia with sodium hydroxide within 22 h and adding glycerol and octanoate in the ratio of 7:3 until 80 g/L accumulation to the fed-batch fermentation method increased the C/N ratio. When the C/N ratio increased, the OD and cell density value also increased until it reached 15 g/L of dried cell density and OD at 122, comparable to the previous report [26]. At the end of a 60-h incubation period, the cells were harvested, lyophilized, and further extracted and characterized. The MCL-PHA polymer that accumulated in the cells was extracted with chloroform (10 mL of chloroform/1 g of dry cells) as described above (Figure 3a), and the wet extracted slurry was white sticky matter (Figure 3b). After allowing the solvent to evaporate at 50 °C, the dried white MCL-PHA could be formed as a transparent film (Figure 3c) by solvent casting. The film tended to have adhesive and elastomeric properties after qualitative evaluation.

### 3.2. Productivity and Monomer Composition of MCL-PHA from Pseudomonas Putida

**Figure 4:**
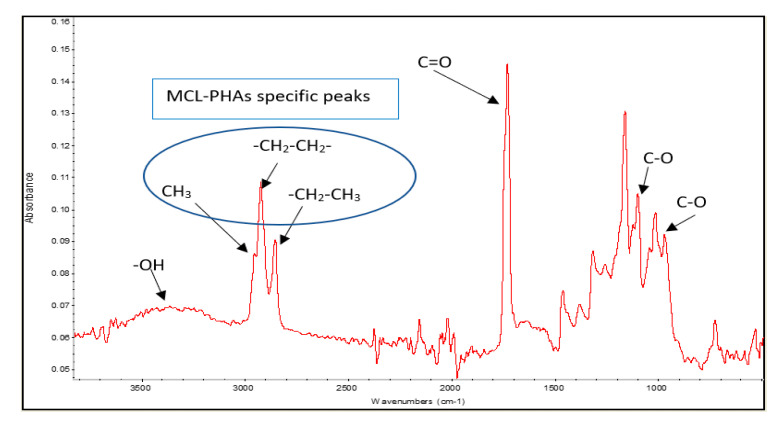
FTIR spectra of MCL-PHA sample.

The polyester biosynthesized by *Pseudomonas putida* ATCC47054 was isolated and purified to determine the PHA content using GC analysis and the PHA monomeric composition by ^1^H-NMR, as listed in Table 1. The PHA productivity and product yield after 60-h cultivation were 0.067 g/L/h and 0.05 g PHA/g substrates, respectively, which is relatively low compared to other reports [27]. The chemical functional group analysis was performed using Fourier Transform Infrared Spectrophotometer (FTIR), as shown in Figure 4. The peaks of the functional groups from FTIR indicated that the produced polymer is MCL-PHA, which exhibits the terminal groups at wavenumbers 2955, 2925, and 2855 cm^−1^. The FTIR spectra show the position of the carbon terminal group, which contains more than three atoms of carbon. They could form a bond to the C-3 position of the hydroxy alkanoic structure, indicating that the synthesized polymers are MCL-PHA [28].

**Figure 5:**
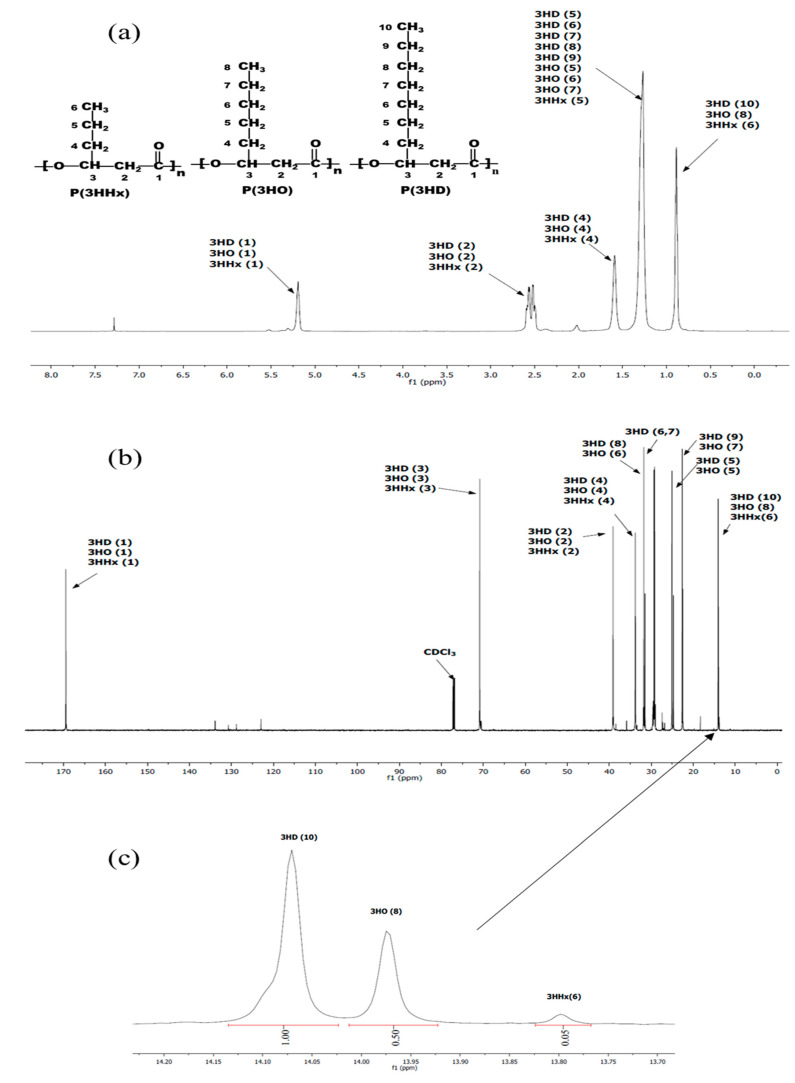
NMR spectra of the extracted MCL-PHA terpolyester sample: (**a**) ^1^H NMR, (**b**) ^13^C NMR and (**c**) enlarged ^13^C NMR spectrum of P(3HHx-*co*-3HO-*co*-3HD) at the terminal carbon group (CH_3_) of 3HD, 3HO, and 3HHx.

The data in the literature suggested that PHA producers growing on fatty acids consist of 8- to 18-carbon synthesize monomers containing 6- to 10-carbon units [29]. The ^1^H and ^13^H NMR spectra (Figure 5a,b) show the position of the spectra indicating the types of MCL-PHA, which consist of 3-hydroxyhexanoate (3HHx), 3-hydroxyoctanoate (3HO), and 3-hydroxydecanoate (3HD). All the signals split into several peaks and were assigned by comparison to previously reported data [30,31]. The enlarged spectrum (Figure 5c) for comparing of the carbon terminal group shows different peaks, at which the positions of 13.80, 13.97, and 14.07 were the terminals of 3HHx, 3HO, and 3HD, respectively [30,32]. After calculation of the relative NMR peak area, the overall chemical composition of the PHA polyester after 60-h cultivation was as follows: 64.5% 3HHx, 32.2% 3HO, and 3.3% 3HD.

A previous study identified the %PHA from the combination of an octanoate source with glycerol as a carbon source (42% of cell dried weight) [33]. The PHA content obtained from this process is higher than the PHA from a single substrate of octanoate. The data also indicate that PHA from octanoate sources contain a 3HO monomer as a main component (80%). The PHA monomer obtained from the combination of glycerol was converted directly to 3HD in the MCL-PHA component, and this study has been entirely consistent with the results. Our biosynthesis results showed that the main monomer composition was 3HD, which may be associated with a higher glycerol ratio than octanoate of the substrate co-feeding. It can be indicated that modifying the carbon source ratio may allow the amount of monomer giving rise to a change in other material properties to be controlled.

### 3.3. Physical Properties of MCL-PHA

**Figure 6:**
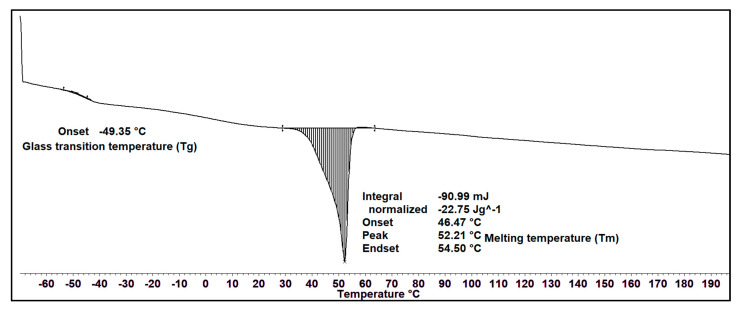
DSC thermogram of MCL-PHA terpolyester sample at the heating rate of 10 °C/min.

The thermal property of the MCL-PHA solvent-cast film was measured using DSC. Figure 6 exhibits the DSC thermograms during the heating scan, observing that the glass transition temperature (*T*_g_) is −49.35 °C, and melting temperature (*T*_m_) is 52.21 °C, which has a melting enthalpy of fusion (∆*H*_m_) of about 22.75 J/g. The crystallinity percentage is equal to ∆*H*_m_/∆*H*_m0_, assuming 146 J/g as the melting enthalpy of 100% crystalline Poly(3-hydroxybutyrate) [34]. The crystallinity percentage of our MCL-PHA terpolyester from this equation is 15.6%. The results of the DSC analysis show the thermal properties of MCL-PHA. The melting temperature and glass transition temperature are similar to the general properties of MCL-PHA [35].

**Figure 7:**
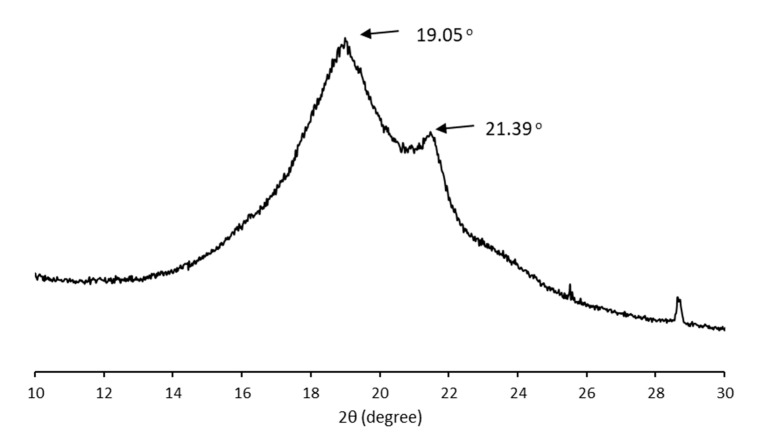
X-ray diffraction pattern of MCL-PHA terpolyester sample.

The crystalline structure of the MCL-PHA sample was characterized using wide-angle X-ray diffraction (WAXD). Figure 7 shows typical X-ray diffraction patterns of solution-cast films from the P(3HHx-co-3HO-co-3HD) terpolyester film. In the initial scan, there is a strong reflection in the group of MCL-PHA, while reflections in the 9–25° range had a little reaction compared to SCL-PHAs. The crystallinity of MCL-PHA calculated from the WAXD result (15.7%) was listed in Table 2 compared to the crystallinity calculated from the DSC result (15.6%). Thus, the WAXD analysis results confirm the crystallinity of MCL-PHA, which can also be detected using DSC, and both of them are almost similar in the percentage of crystallinity.

For this reason, the crystalline structure of MCL-PHA has a high amorphous domain content in the crystallographic structure, causing a low melting temperature of the terpolyester. Strong reflections in the initial angle of the scan clearly show the MCL-PHA. In addition, the peak reflections of X-ray diffraction patterns at 9° indicate the presence of a 3HHx monomer [36]. The crystallinity percentage is 15.6, which indicates the low crystallization of MCL-PHA.

**Figure 8:**
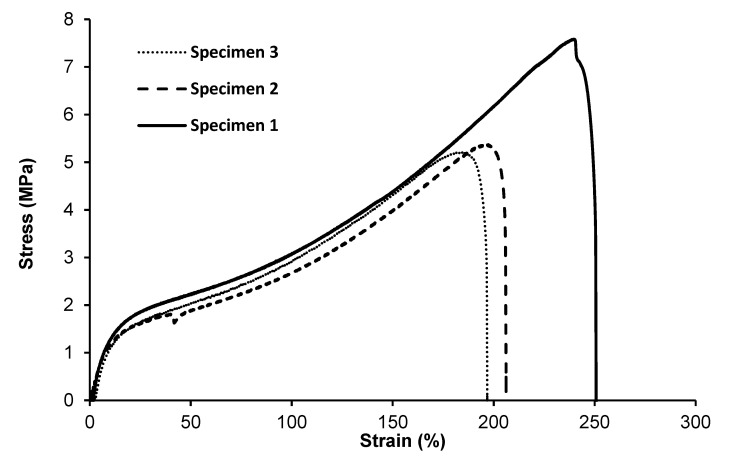
Tensile stress–strain curves of MCL-PHA terpolyester sample. (The repeated 3-time measurements took the data from the three specimens.).

A tensile test was performed to determine the effect of monomer composition on the mechanical properties of MCL-PHA terpolyester films, which were repeatedly tested three times. Test results (Figure 8) show the curves of the relationship between stress and strain. As listed in Table 2, Young’s modulus is 20.07 ± 3.64 MPa, the ultimate strength is 6.05±1.33 MPa, and the elongation at break is 217.85% ± 28.82%, indicating that the molded MCL-PHA film is quite flexible, which is consistent with the exceptional elastomeric characteristics compared with the previous report [16].

Typically, mechanical properties of the MCL-PHA film have Young’s modulus ranged from 1 to 30 MPa, ultimate tensile strength was about 7 MPa, and elongation at break ranged from 250 to 380% [37]. The above-reported values are similar to the physical properties of MCL-PHA from this study, as listed in Table 2. Additionally, considering the stress relationship graph and the percentage elongation, MCL-PHA is an elastomer with flexibility [17]. In addition, MCL-PHA is a stronger elastomer than degradable polyurethane, which makes it attractive for biomedical uses such as tendon and cardiovascular tissue formation [38].

### 3.4. Fabrication Capability and Printability of MCL-PHA

**Figure 9:**
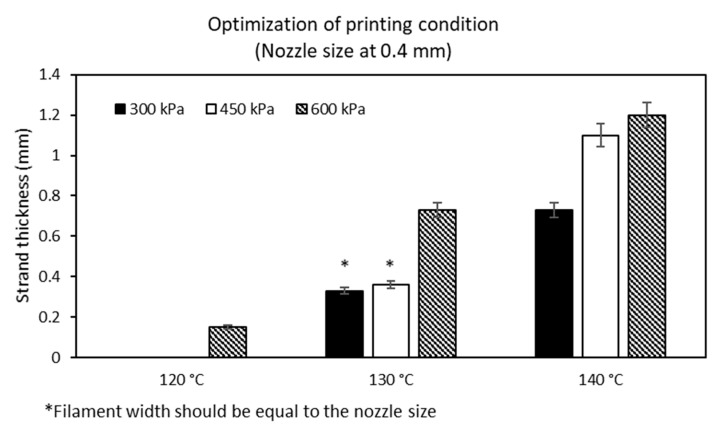
Optimization on printing condition of MCL-PHA terpolyester.

The ideal conditions for printing MCL-PHA were performed by employing a 3D printer. Our study evaluation process was conducted at varying melting temperatures and pressures with a fixed printing speed at 3 mm/s. The above variable parameters affected the filament size (strand thickness), which had to be suitable for the diameter condition of the extrusion nozzle. The optimization results show the relationship between the varied applied temperature conditions at 120, 130, and 140 °C, respectively, and at applied pressures of 300, 450, and 600 kPa (Figure 9). We found that two suitable conditions could be fabricated, consisting of the applied temperature at 130 °C combined with either 300 kPa or 450 kPa of the applied pressures. These two conditions could generate the strand thickness that was closest to the nozzle size of the 3D printer.

**Figure 10:**
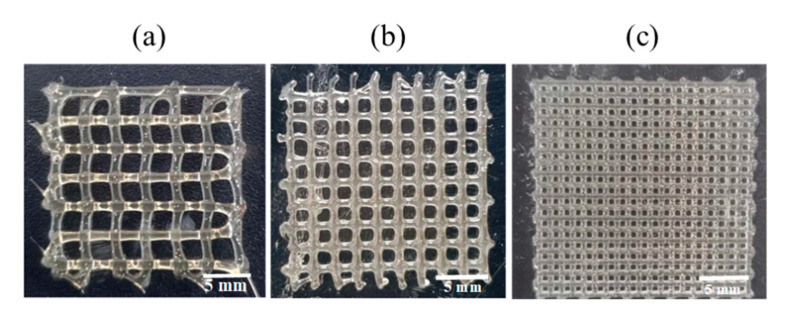
MCL-PHA terpolyester scaffolds fabricated by various conditions (Table 3): (**a**) Condition-1, (**b**) Conditon-2, and (**c**) Condition-3.

Then, the 3D-printing conditions of the MCL-PHA terpolyester scaffolds at an operating temperature of 130 °C and a printing speed at 3 mm/s were evaluated in two conditions of rough printing (0.4 mm printhead) as follows: Condition-1 (Figure 10a), applied pressure at 300 kPa with a 3 × 3 mm^2^ pore size; and Condition-2 (Figure 10b), applied pressure of 450 kPa with a 2 × 2 mm^2^ pore size. The illustrated results in Figure 10a,b, and Table 3 indicated that the Condition-2 scaffold exhibited higher smoothness and consistency, lower strand thickness, and higher printing accuracy than the scaffold of Condition-1. After that, we decreased the printhead diameter to 0.2 mm (fine printing) in Condition-3 (Figure 10c); however, the applied pressure was still fixed at 450 kPa with the same optimal printing operation. As a result, in Figure 10c, the Condition-3 scaffold shows a substantial fine and smooth, consistent printing fiber. Furthermore, as listed in Table 3, the fabricated Condition-3 scaffold has a strand thickness of 0.26 mm, a pore size diameter of 0.36 mm, and a relatively high printing accuracy of 82%. Therefore, it can be concluded that the optimal condition for MCL-PHA printing fabrication is Condition-3, which contains the following three main printing parameters: 0.2 mm printhead, the applied temperature at 130 °C, and applied pressure of 450 kPa. Nevertheless, this printing condition was operated at a substantially high temperature and could not co-print with human cells. Thus, cells should be injected after the scaffold had formed for the biomedical application, similar to the electrospinning technique.

The 3D-printing used in MCL-PHA is rarely studied in other polymers where most other polymers are made using fused deposition modeling (FDM), Selective laser sintering (SLS), and stereolithography (SLA) techniques [39]. The scaffold molded elastomer material using the 3D-printing technique is 3D-plotter silicone, which does not require a high molding temperature [40]. The scaffold molded elastomer material using the 3D-printing technique is 3D-plotter silicone, which does not require high molding temperature, but it is not degradable compared to MCL-PHA. Typically, the factors affecting the 3D-printing fabrication consist of printing pressure and speed, especially in silicone. However, MCL-PHA has a temperature factor that is significantly affected on 3D-printing. Therefore, it is necessary to investigate a temperature that can maintain the viscoelastic state of the MCL-PHA ink, unlike silicone, an elastomer that can be converted to inks without using temperature. The pore size of the moldable MCL-PHA scaffold in our Condition-3 printing fabrication was approximately 0.36 mm, which is in the range of the scaffold pore size suitable for effective cell growth (100–700 μm). This characteristic of pore size strongly depends on the behavior of each cell type used in conjunction with the scaffold [6]. It can be concluded that this research was able to form a microporous MCL-PHA elastomer scaffold and can be employed as a biological elastomer formed using 3D-printing techniques.

### 3.5. Rheological Properties of MCL-PHA Biomaterial-Ink

**Figure 11:**
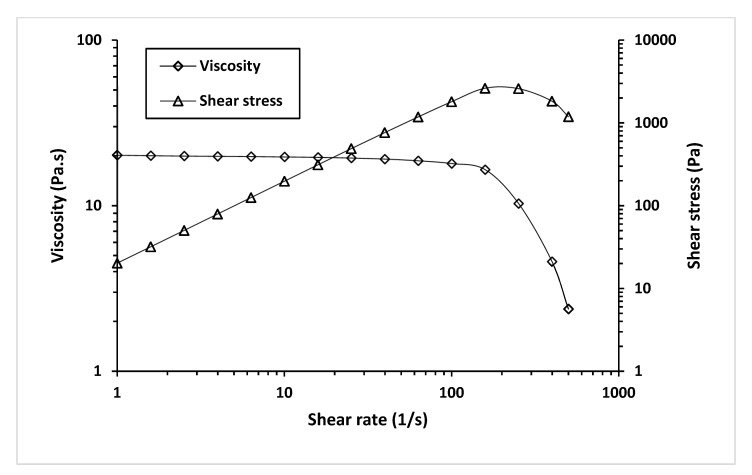
Viscosity and shear stress profiles relative to a shear rate of MCL-PHA biomaterial-ink are rheologically tested at 130 °C.

We investigated the effect of shear rate on the shear stress and viscosity of MCL-PHA biomaterial-ink at an optimized temperature for 3D printing (130 °C), as seen in Figure 11.

Figure 11 exhibits a decrease in viscosity and an increase in shear stress as the shear rate increases. It indicated that shear stress (applied pressure on the material syringe) gave rise to a decrease in the viscosity of the MCL-PHA material. Such behavior falls in a group of non-Newtonian or pseudo-plastic with shear-thinning characteristics [41]. This effect could explain the material behavior when printing from an extruded 3D printer, in which shear forces can overcome the reduced material viscosity, allowing the material to flow out of the nozzle.

**Figure 12:**
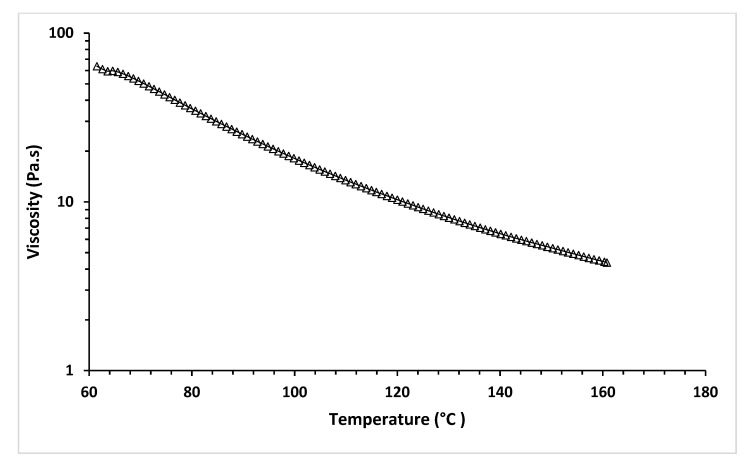
Temperature sweep of rheological test of MCL-PHA biomaterial-ink.

The temperature sweep test of MCL-PHA can exhibit the relationship between temperature and viscosity at a constant shear rate of 18 s^−1^, relating to the applied pressure of 450 kPa of Condition-3. Figure 12 shows the shear stress and temperature sweep viscosity profiles of MCL-PHA biomaterial-ink assumed in the optimized printing condition, leading to good shape fidelity.

Figure 12 shows the viscosity that decreases with increasing temperature. This result indicated the behavior of the material in which it melts and changes from solid to liquid, which causes a decrease in viscosity. The temperature sweep test exhibits the viscosity at 130 °C is at 8.07 Pa·s, indicating that with this condition, a scaffold can be extruded and printed out at the applied pressure over 300 kPa via the 3D printer in this study. This rheological test confirms that material viscosity is a significant parameter in the MCL-PHA material extrusion of 3D-printing techniques [42].

## 4. Conclusions

In this work, MCL-PHA was successfully biosynthesized from co-substrates of glycerol and octanoate via fed-batch fermentation by *Pseudomonas Putida* ATCC 47054 with monomer compositions of 64.5% 3HHx, 32.2% 3HO, and 3.3% 3HD. The MCL-PHA terpolyester has low *T*_m_ (52 °C) and low crystallinity (15.6%), indicating a high ductile amorphous domain in the crystalline structure. The tensile elongation is above 200%, indicating that the MCL-PHA film has an elastomeric property and flexibility. The fabrication of the MCL-PHA scaffold with 3D printing was influenced by controlling the material melting temperature, the polymer extruding pressure, and the printhead diameter. The printed scaffold formation with various conditions resulted in a condition that can form a scaffold with a pore size close to the optimum size for cell proliferation. The rheological property of this biomaterial-ink indicated non-Newtonian fluid behavior. It can be concluded that this study yields a condition that can shape the MCL-PHA to a particular configuration. The customizable MCL-PHA elastomer scaffold can be useful for a wide variety of tissue engineering applications. To the best of our knowledge, this research is the first experiment that reports the utilization of PHA as biomaterial-ink in a 3D-printing fabrication scaffold.

## Figures and Tables

**Table 1:** Biosynthesis of MCL-PHA during 60-h fed-batch cultivation of *Pseudomonas putida*.

Cell Density (g/L)	PHA Content Per Dry Cell ^a^(%)	PHA Yield ^b^ (g/g of substrate)	PHA Productivity (g/L/h)	% PHA Monomer Composition ^c^		
				3HHx	3HO	3HD
15	27	0.05	0.067	3.3 %	32.2 %	64.5%

^a^ PHA content incorporated in cells was determined using GC. ^b^ PHA yield calculated from gram of produced PHA per gram of co-substrates feeding in fermenter. ^c^ PHA monomer composition was determined by 500 MHz ^1^H NMR.

**Table 2:** Physical properties of MCL-PHA terpolyester sample.

Thermal Properties			Crystallinity		Mechanical Properties		
T_g_ (°C)	T_m_ (°C)	∆H_m_ (J/g)	X_c (DSC)_ (%)	X_c (XRD)_ (%)	Young’s Modulus (MPa)	Elongation at Break (%)	Ultimate Strength (MPa)
−49.35	52.21	22.75	15.6 ± 5	15.7 ± 5	20.07 ± 3.64	217.85 ± 28.82	6.05 ± 1.33

**Table 3:** Printing accuracy and fiber characteristics of MCL-PHA terpolyester scaffolds fabricated using 3D-printing at the operation temperature of 130 °C with 3 mm/s of printing speed.

Printing Condition	Applied Pressure (kPa)	Printing Accuracy (%)	Strand Thickness (mm)	Pore Size (mm)
Condition-1, Rough printing (0.4 mm printhead)	300	38%	0.95 ± 0.19	2.87 ± 0.44
Condition-2, Rough printing (0.4 mm printhead)	450	71%	0.49 ± 0.07	1.02 ± 0.03
Condition-3, Fine printing (0.2 mm printhead)	450	82%	0.26 ± 0.04	0.36 ± 0.06

## Data Availability

The data presented in this study are available on request from the corresponding author.
